# 3-{2-[2-(2-Fluoro­benzyl­idene)hydrazin­yl]-1,3-thia­zol-4-yl}-2*H*-chromen-2-one

**DOI:** 10.1107/S1600536810018647

**Published:** 2010-05-26

**Authors:** Afsheen Arshad, Hasnah Osman, Chan Kit Lam, Ching Kheng Quah, Hoong-Kun Fun

**Affiliations:** a School of Chemical Sciences, Universiti Sains Malaysia, 11800 USM, Penang, Malaysia; b School of Pharmaceutical Sciences, Universiti Sains Malaysia, 11800 USM, Penang, Malaysia; c X-ray Crystallography Unit, School of Physics, Universiti Sains Malaysia, 11800 USM, Penang, Malaysia

## Abstract

In the title compound, C_19_H_12_FN_3_O_2_S, the chromene ring system and the thia­zole ring are approximately planar [maximum deviations of 0.023 (3) Å and 0.004 (2) Å, respectively]. The chromene ring system is inclined at angles of 4.78 (10) and 26.51 (10)° with respect to the thia­zole and benzene rings, respectively, while the thia­zole ring makes a dihedral angle of 23.07 (12)° with the benzene ring. The mol­ecular structure is stabilized by an intra­molecular C—H⋯O hydrogen bond, which generates an *S*(6) ring motif. The crystal packing is consolidated by inter­molecular N—H⋯O hydrogen bonds, which link the mol­ecules into chains parallel to [100], and by C—H⋯π and π–π [centroid–centroid distance = 3.4954 (15) Å] stacking inter­actions.

## Related literature

For the synthesis of the title compound, see: Lv *et al.* (2010 ▶); Siddiqui *et al.* (2009 ▶). For general background to and the biological activity of coumarin derivatives, see: Anderson *et al.* (2002 ▶); Tassies *et al.* (2002 ▶); Mitscher (2002 ▶); Lafitte *et al.* (2002 ▶); Moffett (1964 ▶); Weber *et al.* (1998 ▶). For the biological activity of amino­thia­zoles derivatives, see: Hiremath *et al.* (1992 ▶); Habib & Khalil (1984 ▶); Karah *et al.* (1998 ▶); Gursoy & Karah (2000 ▶); Lednicer *et al.* (1990 ▶); Kim *et al.* (2002 ▶); Wattenberg *et al.* (1979 ▶). For the stability of the temperature controller used for the data collection, see: Cosier & Glazer (1986 ▶). For hydrogen-bond motifs, see: Bernstein *et al.* (1995 ▶).
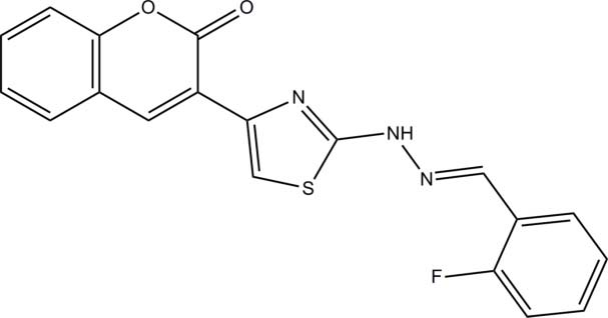


## Experimental

### 

#### Crystal data

C_19_H_12_FN_3_O_2_S
*M* *_r_* = 365.38
Orthorhombic, 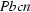

*a* = 12.303 (2) Å
*b* = 10.4477 (17) Å
*c* = 25.247 (4) Å
*V* = 3245.2 (9) Å^3^
*Z* = 8
Mo *K*α radiation
μ = 0.23 mm^−1^
*T* = 100 K
0.37 × 0.08 × 0.04 mm

#### Data collection

Bruker SMART APEXII DUO CCD area-detector diffractometer
Absorption correction: multi-scan (*SADABS*; Bruker, 2009 ▶) *T*_min_ = 0.919, *T*_max_ = 0.991
13589 measured reflections
2855 independent reflections
1971 reflections with *I* > 2σ(*I*)
*R*_int_ = 0.082

#### Refinement

*R*[*F* ^2^ > 2σ(*F* ^2^)] = 0.044
*wR*(*F* ^2^) = 0.102
*S* = 1.04
2855 reflections
239 parameters
H atoms treated by a mixture of independent and constrained refinement
Δρ_max_ = 0.22 e Å^−3^
Δρ_min_ = −0.33 e Å^−3^

### 

Data collection: *APEX2* (Bruker, 2009 ▶); cell refinement: *SAINT* (Bruker, 2009 ▶); data reduction: *SAINT*; program(s) used to solve structure: *SHELXTL* (Sheldrick, 2008 ▶); program(s) used to refine structure: *SHELXTL* ; molecular graphics: *SHELXTL*; software used to prepare material for publication: *SHELXTL* and *PLATON* (Spek, 2009 ▶).

## Supplementary Material

Crystal structure: contains datablocks global, I. DOI: 10.1107/S1600536810018647/tk2674sup1.cif

Structure factors: contains datablocks I. DOI: 10.1107/S1600536810018647/tk2674Isup2.hkl

Additional supplementary materials:  crystallographic information; 3D view; checkCIF report

## Figures and Tables

**Table 1:** Hydrogen-bond geometry (Å, °) *Cg*1 is the centroid of the C13–C18 benzene ring.

*D*—H⋯*A*	*D*—H	H⋯*A*	*D*⋯*A*	*D*—H⋯*A*
C9—H9⋯O2	0.93	2.27	2.823 (3)	118
N2—H12*N*⋯O2^i^	0.85 (3)	2.04 (3)	2.852 (3)	161 (3)
C4—H4⋯*Cg*1^ii^	0.93	2.96	3.701 (3)	138

Symmetry codes: (i) 
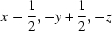
; (ii) 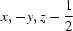.

**Fig. 1.**
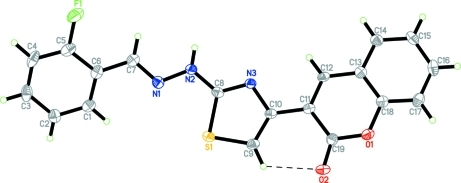
The molecular structure of the title compound, showing 50% probability displacement ellipsoids for non-H atoms and the atom-numbering scheme.The intramolecular C–H···O interaction is shown as a dashed line.

**Fig. 2.**
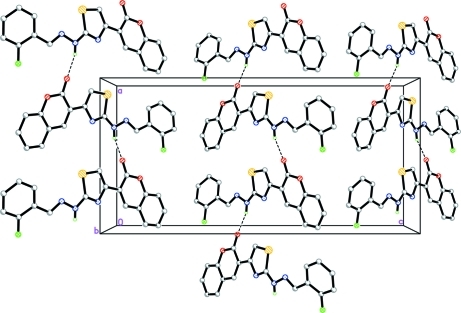
The crystal structure of the title compound viewed along the b axis. H atoms not involved in intermolecular interactions (dashed lines) have been omitted for clarity.

## supplementary crystallographic information

### Comment

Coumarin derivatives constitute an important class of heterocyclic compounds having pronounced biological activities. For example, warfarin and cenocoumarol are used as anti-coagulants (Anderson *et al.*, 2002; Tassies *et al.*, 2002). These compounds also possess very good anti-bacterial (Mitscher, 2002; Lafitte *et al.*, 2002), anti-fungal (Moffett, 1964) and cytotoxic activities (Weber *et al.*, 1998). On the other hand, aminothiazole derivatives have been reported to exhibit significant anti-fungal (Hiremath *et al.*, 1992), anti-bacterial (Habib & Khalil, 1984), and anti-tuberculosis activities (Karah *et al.*, 1998; Gursoy & Karah, 2000). These compounds also have very important pharmaceutical value because of their anti-inflammatory (Lednicer *et al.*, 1990), enzyme inhibition (Kim *et al.*, 2002) and anti-tumour activities (Wattenberg *et al.*, 1979). Our approach is the synthesis of biologically active compounds based on the combination of different substructures to enhance the biological activity of known compounds. The title compound is a new coumarin derivative having aminothiazole moiety. We present here its crystal structure, Fig. 1.

The chromene (O1/C11–C19) ring system and thiazole (S1/N3/C8–C10) ring are approximately planar, with the maximum deviation of 0.023 (3) Å for atom C19 and 0.004 (2) Å for atom N3, respectively. The chromene ring system is inclined at angles of 4.78 (10) and 26.51 (10) ° with respect to the thiazole and benzene (C1–C6) rings, respectively. The thiazole ring makes a dihedral angle of 23.07 (12) ° with benzene ring. The molecular structure is stabilized by an intramolecular C9—H9···O2 hydrogen bond which generates an *S*(6) ring motif (Bernstein *et al.*, 1995).

The crystal packing is consolidated by intermolecular N2—H12N···O2 hydrogen bonds (Fig. 2) which link the independent molecules into chains parallel to [1 0 0]. The crystal packing is consolidated by C—H···π (Table 1) and π–π stacking interactions between symmetry related S1/N3/C8—C10 (centroid Cg2) and O1/C11—C13/C18/C19 (centroid Cg3) rings, with Cg2···Cg3 distance of 3.4954 (15) Å [symmetry code: 3/2-x, -1/2+y, z].

### Experimental

Thiosemicarbazide (5.00 mmol) was slowly added to a solution of 2-fluorobenzaldehyde in hot absolute ethanol (10 ml) while stirring. The resulting solution was refluxed for 2 h and then cooled on an ice bath for 45 minutes to get white precipitates. These precipitates were filtered and recrystallized from ethanol-water to obtain 2-fluorobenzaldehyde thiosemicarbazone (Lv *et al.*, 2010). 3-[ω-Bromoacetyl coumarin] was prepared as reported in the literature (Siddiqui *et al.*, 2009). A solution of 3-[ω-bromoacetyl coumarin] (2.50 mmol) and 2-fluorobenzaldehyde thiosemicarbazone (2.50 mmol) in chloroform-ethanol (2:1) was refluxed for 30 min. Precipitates formed were filtered and boiled in water containing sodium acetate. The product was purified by recrystallization with ethanol-chloroform (8:2).

### Refinement

Atom H12N was located in a difference Fourier map and allowed to refined freely. The remaining hydrogen atoms were positioned geometrically and refined using a riding model with C—H = 0.93 Å and U_iso_(H) = 1.2 U_eq_(C).

### Crystal data

**Table:**

C_19_H_12_FN_3_O_2_S	*F*(000) = 1504
*M**_r_* = 365.38	*D*_x_ = 1.496 Mg m^−^^3^
Orthorhombic, *P**b**c**n*	Mo *K*α radiation, λ = 0.71073 Å
Hall symbol: -P 2n 2ab	Cell parameters from 1466 reflections
*a* = 12.303 (2) Å	θ = 3.2–27.1°
*b* = 10.4477 (17) Å	µ = 0.23 mm^−^^1^
*c* = 25.247 (4) Å	*T* = 100 K
*V* = 3245.2 (9) Å^3^	Needle, yellow
*Z* = 8	0.37 × 0.08 × 0.04 mm

### Data collection

**Table:**

Bruker SMART APEXII DUO CCD area-detector diffractometer	2855 independent reflections
Radiation source: fine-focus sealed tube	1971 reflections with *I* > 2σ(*I*)
graphite	*R*_int_ = 0.082
φ and ω scans	θ_max_ = 25.0°, θ_min_ = 1.6°
Absorption correction: multi-scan (*SADABS*; Bruker, 2009)	*h* = −12→14
*T*_min_ = 0.919, *T*_max_ = 0.991	*k* = −12→12
13589 measured reflections	*l* = −25→30

### Refinement

**Table:**

Refinement on *F*^2^	Primary atom site location: structure-invariant direct methods
Least-squares matrix: full	Secondary atom site location: difference Fourier map
*R*[*F*^2^ > 2σ(*F*^2^)] = 0.044	Hydrogen site location: inferred from neighbouring sites
*wR*(*F*^2^) = 0.102	H atoms treated by a mixture of independent and constrained refinement
*S* = 1.04	*w* = 1/[σ^2^(*F*_o_^2^) + (0.0409*P*)^2^ + 0.4898*P*] where *P* = (*F*_o_^2^ + 2*F*_c_^2^)/3
2855 reflections	(Δ/σ)_max_ < 0.001
239 parameters	Δρ_max_ = 0.22 e Å^−^^3^
0 restraints	Δρ_min_ = −0.33 e Å^−^^3^

### Special details

**Table:**

Experimental. The crystal was placed in the cold stream of an Oxford Cyrosystems Cobra open-flow nitrogen cryostat (Cosier & Glazer, 1986) operating at 100.0 (1) K.
Geometry. All esds (except the esd in the dihedral angle between two l.s. planes) are estimated using the full covariance matrix. The cell esds are taken into account individually in the estimation of esds in distances, angles and torsion angles; correlations between esds in cell parameters are only used when they are defined by crystal symmetry. An approximate (isotropic) treatment of cell esds is used for estimating esds involving l.s. planes.
Refinement. Refinement of *F*^2^ against ALL reflections. The weighted *R*-factor wR and goodness of fit *S* are based on *F*^2^, conventional *R*-factors *R* are based on *F*, with *F* set to zero for negative *F*^2^. The threshold expression of *F*^2^ > σ(*F*^2^) is used only for calculating *R*-factors(gt) etc. and is not relevant to the choice of reflections for refinement. *R*-factors based on *F*^2^ are statistically about twice as large as those based on *F*, and *R*- factors based on ALL data will be even larger.

### Fractional atomic coordinates and isotropic or equivalent isotropic displacement parameters (Å^2^)

**Table:**

	*x*	*y*	*z*	*U*_iso_*/*U*_eq_	
S1	0.88953 (5)	0.06999 (6)	0.02651 (3)	0.02149 (19)	
F1	0.54027 (12)	−0.24735 (15)	0.18957 (7)	0.0376 (5)	
O1	0.85525 (13)	0.52077 (17)	−0.12371 (7)	0.0230 (5)	
O2	0.97044 (14)	0.41533 (18)	−0.07423 (8)	0.0309 (5)	
N1	0.72862 (17)	−0.0844 (2)	0.07751 (9)	0.0205 (5)	
N2	0.68221 (19)	0.0019 (2)	0.04366 (9)	0.0211 (5)	
N3	0.71706 (16)	0.17627 (19)	−0.01285 (9)	0.0179 (5)	
C1	0.8140 (2)	−0.2887 (2)	0.14056 (11)	0.0225 (6)	
H1	0.8600	−0.2571	0.1144	0.027*	
C2	0.8520 (2)	−0.3806 (2)	0.17528 (11)	0.0244 (7)	
H2	0.9232	−0.4097	0.1725	0.029*	
C3	0.7845 (2)	−0.4299 (3)	0.21429 (11)	0.0293 (7)	
H3	0.8099	−0.4933	0.2370	0.035*	
C4	0.6792 (2)	−0.3843 (3)	0.21930 (11)	0.0296 (7)	
H4	0.6334	−0.4155	0.2456	0.035*	
C5	0.6442 (2)	−0.2922 (3)	0.18451 (12)	0.0258 (7)	
C6	0.7077 (2)	−0.2424 (2)	0.14401 (11)	0.0214 (6)	
C7	0.6640 (2)	−0.1475 (2)	0.10749 (11)	0.0210 (6)	
H7	0.5895	−0.1329	0.1060	0.025*	
C8	0.7499 (2)	0.0851 (2)	0.01854 (10)	0.0181 (6)	
C9	0.9051 (2)	0.1990 (2)	−0.01532 (11)	0.0216 (6)	
H9	0.9717	0.2340	−0.0250	0.026*	
C10	0.80743 (19)	0.2424 (2)	−0.03219 (10)	0.0169 (6)	
C11	0.78532 (19)	0.3487 (2)	−0.06865 (11)	0.0176 (6)	
C12	0.6839 (2)	0.3765 (2)	−0.08582 (10)	0.0181 (6)	
H12	0.6256	0.3283	−0.0735	0.022*	
C13	0.6639 (2)	0.4783 (2)	−0.12245 (11)	0.0197 (6)	
C14	0.5599 (2)	0.5110 (3)	−0.14162 (11)	0.0225 (6)	
H14	0.4993	0.4657	−0.1300	0.027*	
C15	0.5474 (2)	0.6092 (2)	−0.17731 (11)	0.0244 (7)	
H15	0.4785	0.6302	−0.1897	0.029*	
C16	0.6378 (2)	0.6774 (3)	−0.19495 (11)	0.0264 (7)	
H16	0.6288	0.7433	−0.2193	0.032*	
C17	0.7405 (2)	0.6480 (2)	−0.17669 (11)	0.0243 (7)	
H17	0.8008	0.6940	−0.1881	0.029*	
C18	0.7515 (2)	0.5489 (2)	−0.14105 (11)	0.0197 (6)	
C19	0.8764 (2)	0.4266 (2)	−0.08730 (11)	0.0206 (6)	
H12N	0.615 (2)	0.019 (3)	0.0458 (11)	0.030 (8)*	

### Atomic displacement parameters (Å^2^)

**Table:**

	*U*^11^	*U*^22^	*U*^33^	*U*^12^	*U*^13^	*U*^23^
S1	0.0159 (3)	0.0227 (4)	0.0259 (4)	0.0030 (3)	−0.0001 (3)	0.0013 (3)
F1	0.0327 (10)	0.0392 (10)	0.0409 (12)	−0.0040 (8)	0.0141 (8)	0.0027 (9)
O1	0.0179 (10)	0.0250 (10)	0.0261 (12)	−0.0044 (8)	0.0005 (8)	0.0017 (9)
O2	0.0147 (10)	0.0368 (11)	0.0413 (14)	−0.0049 (9)	−0.0028 (9)	0.0075 (10)
N1	0.0232 (12)	0.0150 (11)	0.0233 (14)	0.0024 (10)	0.0011 (10)	−0.0016 (10)
N2	0.0160 (12)	0.0197 (12)	0.0277 (15)	0.0023 (11)	0.0025 (10)	0.0032 (11)
N3	0.0142 (11)	0.0176 (11)	0.0219 (14)	0.0009 (9)	0.0013 (9)	0.0004 (10)
C1	0.0245 (15)	0.0192 (13)	0.0237 (17)	−0.0059 (12)	0.0010 (12)	−0.0045 (13)
C2	0.0299 (16)	0.0183 (14)	0.0251 (18)	−0.0026 (12)	−0.0030 (13)	−0.0016 (13)
C3	0.0457 (19)	0.0181 (14)	0.0240 (18)	−0.0078 (14)	−0.0051 (13)	0.0032 (14)
C4	0.0436 (19)	0.0239 (15)	0.0212 (18)	−0.0147 (14)	0.0058 (14)	0.0001 (13)
C5	0.0262 (16)	0.0240 (15)	0.0273 (18)	−0.0067 (13)	0.0053 (13)	−0.0072 (14)
C6	0.0290 (15)	0.0144 (13)	0.0207 (17)	−0.0052 (12)	0.0006 (12)	−0.0050 (12)
C7	0.0242 (15)	0.0159 (13)	0.0228 (17)	−0.0007 (12)	0.0038 (12)	−0.0051 (12)
C8	0.0171 (13)	0.0158 (12)	0.0213 (16)	0.0024 (11)	−0.0012 (11)	−0.0051 (12)
C9	0.0170 (14)	0.0227 (14)	0.0252 (17)	0.0010 (12)	0.0042 (12)	−0.0024 (12)
C10	0.0148 (13)	0.0177 (12)	0.0183 (15)	−0.0012 (11)	−0.0001 (11)	−0.0049 (12)
C11	0.0165 (14)	0.0175 (13)	0.0189 (16)	−0.0013 (11)	0.0017 (11)	−0.0044 (12)
C12	0.0153 (13)	0.0189 (13)	0.0202 (16)	−0.0009 (11)	0.0040 (11)	−0.0034 (12)
C13	0.0218 (15)	0.0182 (13)	0.0191 (16)	0.0009 (11)	0.0026 (11)	−0.0040 (12)
C14	0.0219 (15)	0.0242 (15)	0.0215 (17)	0.0026 (12)	0.0023 (12)	−0.0015 (13)
C15	0.0263 (16)	0.0250 (15)	0.0220 (18)	0.0087 (13)	−0.0023 (12)	−0.0016 (13)
C16	0.0399 (18)	0.0188 (14)	0.0206 (18)	0.0042 (13)	−0.0005 (13)	0.0003 (13)
C17	0.0310 (16)	0.0208 (14)	0.0210 (17)	−0.0051 (13)	0.0027 (13)	−0.0020 (13)
C18	0.0211 (14)	0.0175 (13)	0.0206 (16)	0.0002 (11)	0.0007 (12)	−0.0051 (12)
C19	0.0214 (15)	0.0183 (13)	0.0222 (16)	−0.0009 (12)	−0.0004 (11)	−0.0010 (13)

### Geometric parameters (Å, °)

**Table:**

S1—C9	1.723 (3)	C5—C6	1.388 (4)
S1—C8	1.736 (2)	C6—C7	1.456 (4)
F1—C5	1.367 (3)	C7—H7	0.9300
O1—C19	1.372 (3)	C9—C10	1.354 (3)
O1—C18	1.381 (3)	C9—H9	0.9300
O2—C19	1.209 (3)	C10—C11	1.469 (4)
N1—C7	1.281 (3)	C11—C12	1.353 (3)
N1—N2	1.368 (3)	C11—C19	1.462 (4)
N2—C8	1.361 (3)	C12—C13	1.431 (4)
N2—H12N	0.85 (3)	C12—H12	0.9300
N3—C8	1.303 (3)	C13—C18	1.387 (4)
N3—C10	1.397 (3)	C13—C14	1.410 (4)
C1—C2	1.382 (4)	C14—C15	1.374 (4)
C1—C6	1.397 (4)	C14—H14	0.9300
C1—H1	0.9300	C15—C16	1.394 (4)
C2—C3	1.387 (4)	C15—H15	0.9300
C2—H2	0.9300	C16—C17	1.379 (4)
C3—C4	1.386 (4)	C16—H16	0.9300
C3—H3	0.9300	C17—C18	1.379 (4)
C4—C5	1.372 (4)	C17—H17	0.9300
C4—H4	0.9300		
			
C9—S1—C8	88.17 (12)	C10—C9—H9	124.6
C19—O1—C18	122.7 (2)	S1—C9—H9	124.6
C7—N1—N2	116.7 (2)	C9—C10—N3	115.5 (2)
C8—N2—N1	117.2 (2)	C9—C10—C11	128.0 (2)
C8—N2—H12N	119.4 (19)	N3—C10—C11	116.5 (2)
N1—N2—H12N	121 (2)	C12—C11—C19	119.0 (2)
C8—N3—C10	109.1 (2)	C12—C11—C10	122.3 (2)
C2—C1—C6	121.2 (3)	C19—C11—C10	118.7 (2)
C2—C1—H1	119.4	C11—C12—C13	121.7 (2)
C6—C1—H1	119.4	C11—C12—H12	119.2
C1—C2—C3	120.4 (3)	C13—C12—H12	119.2
C1—C2—H2	119.8	C18—C13—C14	117.4 (2)
C3—C2—H2	119.8	C18—C13—C12	118.7 (2)
C4—C3—C2	119.8 (3)	C14—C13—C12	123.9 (2)
C4—C3—H3	120.1	C15—C14—C13	120.5 (2)
C2—C3—H3	120.1	C15—C14—H14	119.8
C5—C4—C3	118.4 (3)	C13—C14—H14	119.8
C5—C4—H4	120.8	C14—C15—C16	120.1 (2)
C3—C4—H4	120.8	C14—C15—H15	120.0
F1—C5—C4	118.3 (2)	C16—C15—H15	120.0
F1—C5—C6	117.8 (3)	C17—C16—C15	120.7 (3)
C4—C5—C6	123.9 (3)	C17—C16—H16	119.6
C5—C6—C1	116.3 (3)	C15—C16—H16	119.6
C5—C6—C7	120.9 (2)	C16—C17—C18	118.4 (3)
C1—C6—C7	122.8 (3)	C16—C17—H17	120.8
N1—C7—C6	119.7 (2)	C18—C17—H17	120.8
N1—C7—H7	120.2	C17—C18—O1	117.2 (2)
C6—C7—H7	120.2	C17—C18—C13	122.9 (2)
N3—C8—N2	124.1 (2)	O1—C18—C13	119.8 (2)
N3—C8—S1	116.36 (19)	O2—C19—O1	115.7 (2)
N2—C8—S1	119.6 (2)	O2—C19—C11	126.3 (2)
C10—C9—S1	110.85 (19)	O1—C19—C11	118.0 (2)
			
C7—N1—N2—C8	168.7 (2)	N3—C10—C11—C12	−4.5 (4)
C6—C1—C2—C3	0.5 (4)	C9—C10—C11—C19	−4.9 (4)
C1—C2—C3—C4	−1.6 (4)	N3—C10—C11—C19	176.0 (2)
C2—C3—C4—C5	1.0 (4)	C19—C11—C12—C13	1.2 (4)
C3—C4—C5—F1	−179.9 (2)	C10—C11—C12—C13	−178.3 (2)
C3—C4—C5—C6	0.7 (4)	C11—C12—C13—C18	0.7 (4)
F1—C5—C6—C1	178.9 (2)	C11—C12—C13—C14	179.9 (2)
C4—C5—C6—C1	−1.7 (4)	C18—C13—C14—C15	0.0 (4)
F1—C5—C6—C7	−1.3 (4)	C12—C13—C14—C15	−179.2 (3)
C4—C5—C6—C7	178.1 (2)	C13—C14—C15—C16	0.1 (4)
C2—C1—C6—C5	1.0 (4)	C14—C15—C16—C17	−0.5 (4)
C2—C1—C6—C7	−178.7 (2)	C15—C16—C17—C18	0.7 (4)
N2—N1—C7—C6	179.2 (2)	C16—C17—C18—O1	179.5 (2)
C5—C6—C7—N1	166.1 (2)	C16—C17—C18—C13	−0.6 (4)
C1—C6—C7—N1	−14.2 (4)	C19—O1—C18—C17	178.3 (2)
C10—N3—C8—N2	−179.4 (2)	C19—O1—C18—C13	−1.6 (4)
C10—N3—C8—S1	−0.9 (3)	C14—C13—C18—C17	0.3 (4)
N1—N2—C8—N3	−176.7 (2)	C12—C13—C18—C17	179.5 (2)
N1—N2—C8—S1	4.9 (3)	C14—C13—C18—O1	−179.8 (2)
C9—S1—C8—N3	0.9 (2)	C12—C13—C18—O1	−0.6 (4)
C9—S1—C8—N2	179.4 (2)	C18—O1—C19—O2	−177.4 (2)
C8—S1—C9—C10	−0.5 (2)	C18—O1—C19—C11	3.5 (3)
S1—C9—C10—N3	0.1 (3)	C12—C11—C19—O2	177.7 (3)
S1—C9—C10—C11	−179.0 (2)	C10—C11—C19—O2	−2.7 (4)
C8—N3—C10—C9	0.5 (3)	C12—C11—C19—O1	−3.3 (4)
C8—N3—C10—C11	179.7 (2)	C10—C11—C19—O1	176.3 (2)
C9—C10—C11—C12	174.6 (3)		

### Hydrogen-bond geometry (Å, °)

**Table:**

Cg1 is the centroid of the C13–C18 benzene ring.

**Table:**

*D*—H···*A*	*D*—H	H···*A*	*D*···*A*	*D*—H···*A*
C9—H9···O2	0.93	2.27	2.823 (3)	118
N2—H12N···O2^i^	0.85 (3)	2.04 (3)	2.852 (3)	161 (3)
C4—H4···Cg1^ii^	0.93	2.96	3.701 (3)	138

Symmetry codes: (i) *x*−1/2, −*y*+1/2, −*z*; (ii) *x*, −*y*, *z*−1/2.
